# Joint Imbalance Adaptation for Radiology Report Generation

**DOI:** 10.1007/s41666-025-00205-9

**Published:** 2025-06-20

**Authors:** Wang Li, Guangzeng Han, Yuexin Wu, I.-Chan Huang, Xiaolei Huang

**Affiliations:** 1 Department of Computer Science, University of Memphis, Memphis, TN 38152 USA; 2 Department of Epidemiology and Cancer Control, St. Jude Children’s Research Hospital, Memphis, TN 38105 USA

**Keywords:** Data imbalance, Radiology report generation, Curriculum learning, Model robustness

## Abstract

Radiology report generation, translating radiological images into precise and clinically relevant description, may face the data imbalance challenge — medical tokens appear less frequently than regular tokens, and normal entries are significantly more than abnormal ones. However, very few studies consider the imbalance issues, not even with conjugate imbalance factors. In this study, we propose a **J**oint **Im**balance **A**daptation (*JIMA*) model to promote task robustness by leveraging token and label imbalance. We employ a hard-to-easy learning strategy that mitigates overfitting to frequent labels and tokens, thereby encouraging the model to focus more on infrequent labels and clinical tokens. JIMA presents notable improvements (16.75–50.50% on average) across evaluation metrics on IU X-ray and MIMIC-CXR datasets. Our ablation analysis and human evaluations show the improvements mainly come from enhancing performance on infrequent tokens and abnormal radiological entries, which can also lead to more clinically accurate reports. While data imbalance (e.g., infrequent tokens and abnormal labels) can lead to the underperformance of radiology report generation, our imbalance learning strategy opens promising directions on how to encounter data imbalance by reducing overfitting on frequent patterns and underfitting on infrequent patterns.

## Introduction

**Fig. 1:**
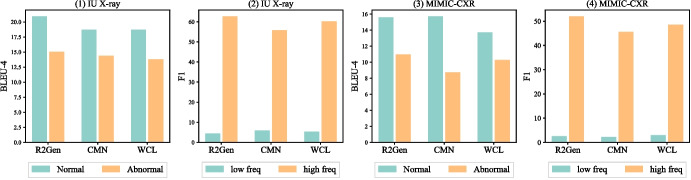
State-of-the-art model performance on normal and abnormal entries by BLEU-4 (left two) and low- and high-frequent tokens by F1 scores (right two). We used two different colors to denote model performance on normal (orange) vs abnormal (light green) reports or frequent (orange) vs infrequent (light green) tokens

Radiology report generation is a multimodal and medical image-to-text task that generates text descriptions for radiographs (e.g., X-ray or CT scan), which may reduce the workloads of radiologists [1, 2]. The task has own unique characteristics than general image-to-text tasks (e.g., image captioning), such as lengthy medical notes, medical annotations, and clinical terminologies. As demonstrated in Fig. 1, *data imbalance* can significantly impact model robustness that prevents model deployment in practice — models can easily overfit on frequent patterns. However, modeling data imbalance to augment the robust generation of the radiology report is understudied.

Two major data imbalances exist in the radiology generation task, label and token. *Label imbalance* pertains to a disproportionate ratio of normal and abnormal diagnosis categories, which exist in radiological images and text reports. For instance, normal cases (images and reports) dominate radiology data, which can easily lead to underperformance in disease detection and professional description. As shown in Table 1, abnormal reports are considerably longer than normal reports while can only count less than 15%. These abnormal reports are much harder to generate than shorter reports [3–5] and can be worse with fewer samples than normal cases.^1^ Existing imbalance learning studies of radiology report generation primarily focus on label imbalance [7, 8]. *Token imbalance* is a critical challenge in generation that tokens have varied occurrence frequencies, and the issue is more critical in the medical task. Learning infrequent tokens can be harder than frequent tokens for generation models [9, 10]. Medical tokens appear less frequently than regular ones, and the infrequent tokens may contain more medical results, highlighting the very unique challenge of this task. The imbalance issue in radiology report generation also connects to broader challenges in multi-label classification or image object detection tasks, where models often struggle to effectively learn from infrequent but clinically significant labels [11] and object categories [12]. Figure 1 illustrates the learning progress of the state-of-the-art (SOTA) model RRG [13] in predicting a report with predominantly normal diagnoses. The model shows strong performance on normal cases but struggles on abnormal reports.

**Table 1:** Data statistics summary

	Image	Report	Vocab	Abnormal %	Normal %	*L*	$L_{normal}$	$L_{abnormal}$
IU X-ray	7470	3955	1517	32.96%	67.04%	35.99	27.76	40.72
MIMIC-CXR	377,110	227,835	13,876	13.97%	86.03%	59.70	34.57	59.36

Variations exist in label (Normal and Abnormal %) and average report length (*L*). Vocab refers to the vocabular size, and normal or abnormal indicates report labels

To promote the quality of generated reports, we propose **J**oint **Im**balance **A**daptation (JIMA) model by curriculum learning [14], a training strategy to present models with increasingly complex examples and mimic human learning from simple to difficult task. JIMA automatically guides the model learning process by leveraging optimization difficulties, strengthening learning capability on infrequent samples, and alleviating overfitting on frequent patterns on both label and token. Our method implements a tailored curriculum strategy that dynamically adjusts data example difficulties by integrating radiology-domain-specific knowledge and difficulty metrics not previously considered in curriculum learning approaches, which aims to address data imbalance and promote radiology report quality. We incorporate the token and label metrics as a joint optimization and design a novel Training Scheduler that sampling and sorting training instances with a multi-aspect scoring mechanism. The scheduler automatically adjusts training samples when model performance varies across multiple imbalance factors. We conduct experiments on two publicly available datasets, MIMIC-CXR [15] and IU X-ray [16] with automatic and human evaluations. By comparing with six state-of-the-art (SOTA) baselines on overall and imbalance performance settings, our approach shows promising results over the SOTA baselines. Notably, JIMA demonstrates minimal impact on the performance of highly frequent tokens and labels, significantly enhances performance for moderately frequent samples, and reveals limitations in boosting performance for the rarest tokens. Our ablation and qualitative analyses show that JIMA can generate more precise medical reports, alleviating label and token imbalance.

## Related Work

**Radiology report generation** is a domain-specific image-to-text task that has two major directions, retrieval- [17, 18] and generation-based [19–21]. The retrieval-based approach compares similarities between an input radiology image and a set of report candidates, ranks the candidates, and returns the most similar one [5, 17, 18, 22, 23]. In contrast, our study focuses on the generation-based task, which automatically generates a precise report from an input image. The task has domain-specific characteristics in the clinical field. The clinical data contains many infrequent medical terminologies and longer documents than image captioning from general domains [6]. As radiology report generation can reduce the workloads of radiologists, generating highly qualified and precise can be a critical challenge, especially under the imbalance settings. Differing from previous work, we aim to promote model robustness and reliability under imbalance settings, which have been rarely studied in the radiology report generation.

**Imbalance learning** aims to model skewed data distributions. The primary focus of imbalance learning is on class or label imbalance, such as positive or negative reviews in sentiment analysis [11]. Recent studies have proposed new approaches to solve imbalance issues by weighting hard and infrequent examples [12] or leveraging imbalance distributions to augment minority data labels [11, 24]. The studies can inspire our work to model multiple imbalance factors, as the imbalance is a multifaceted issue in radiology report generation that exists beyond the data label. Some studies developed new objective functions or data augmentation approach to promote model performance on minority labels, such as focal-loss by down-weighting easy samples [12] or SMOTE by creating synthetic data on minority labels [24]. However, those methods may not be applicable to our primary generation unit, token, which has large vocabulary sizes and extreme sparsity. In terms of radiology report generation, reports may have disease-related labels. Recent studies have augmented model robustness by balancing performance between disease and normal by reinforcement learning [7, 8]. However, those methods focused on the label imbalance, and our study considers a multifaceted imbalance challenge, including label and token imbalance. The token imbalance can be even more critical for the clinical domain, as medical tokens appear less frequently than regular tokens in radiology reports. A close work is the TIMER [10], which considers the token imbalance. However, the approach ignores other imbalance factors, which is solved by our approach. Particularly, our approach jointly models multiple imbalance factors, label and token, and we propose a new curriculum learning method to learn the imbalance factors.

**Fig. 2:**
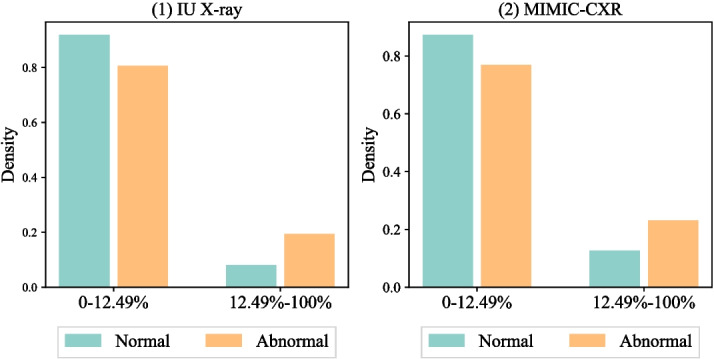
Frequent and infrequent token distributions conditioning on report label. We denote the report types with light green (normal) and orange (abnormal), which show different token imbalance distributions

## Data

We collected two publicly accessible datasets for this study, IU X-ray [16] and MIMIC-CXR [15], de-identified chest X-ray datasets to evaluate radiology report generation. IU X-ray [16], collected from the Indiana Network for Patient Care, includes 7470 X-ray images and corresponding 3955 radiology reports. MIMIC-CXR [15], collected from the Beth Israel Deaconess Medical Center, contains 377,110 X-ray images and 227,827 radiology reports for 65,379 patients. Each report is a text document and associates with one or more front and side X-ray images. Table 1 summarizes statistics of data imbalance and Fig. 2 visualizes the distributions of frequent (ranked in the top 12.5% of the vocabulary) and infrequent tokens. We include preprocessing details in Appendix 1.

Table 1 presents imbalance patterns in tokens and labels. Abnormal entries are predominant in both datasets, and MIMIC-CXR displays a more skewed label distribution, as more abnormal samples were collected during diagnosis phases not for screening purposes. MIMIC-CXR has a longer average length than IU X-ray. The lengthier documents may pose a unique multimodal generation challenge in the medical field. To conduct our analysis, we define the low and high frequencies using the top 12.5% frequent tokens. Figure 1 suggests a joint relation between label and token imbalance and higher ratios of low-frequency tokens in abnormal reports. This observation motivates us to investigate how the imbalance impacts model robustness and reliability.

### Imbalance Effects

We examine the potential impact of label and token imbalance on model performance. To ensure consistency, we keep the top 12.5% to split low- and high-frequent tokens for evaluation purposes. The analysis includes three state-of-the-art models, R2Gen [19], WCL [25], and CMN [26]. We use BLEU-4 [27] and F1 scores to measure performance across both token (low vs high frequency) and label (normal vs. abnormal) imbalance. We visualize performance variations in Fig. 2.

The results suggest that the models exhibit significant difficulties in coping under label and token imbalance. Models consistently perform worse on abnormal reports, which are lengthier and have more infrequent tokens than normal reports. For example, the top 12.5% frequent tokens count > 80% tokens in two datasets, and low-frequent tokens have much worse performance than frequent tokens, as infrequent tokens are harder to optimize [28]. However, infrequent tokens contain higher ratios of medical terms (e.g., silhouettes and pulmonary) describing health states. The significantly varying performance highlights the unique challenges to adapt token and label imbalance. While existing work [7] has considered label imbalance, however, the study did not examine the performance effects of label or token imbalance. The findings inspire us to propose our model **J**oint **Im**balance **A**daptation (*JIMA*) to model token and label imbalance.

## Joint Imbalance Adaptation

**Fig. 3:**
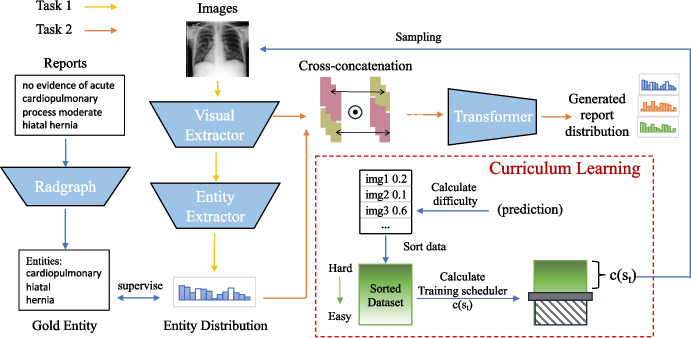
JIMA has two curriculum learning tasks. Task 1 aims to predict entity distribution from images, and task 2 aims to generate a report from the image’s feature and entity distribution. We assign one color per task and solid arrows as workflows. We extracted imbalanced entity distributions from the training data by the Radgraph as the gold truth and compared the entity estimation with the predicted entity distribution. We feed the fused radiology image features and imbalance patterns for the generation process. During the training, tasks 1 and 2 decide which data samples will be fed for the model training

In this section, we present our approach **J**oint **Im**balance **A**daptation (*JIMA*) in Fig. 3 by using *curriculum learning*. JIMA aims to augment model robustness under label and token imbalance. As optimizing data imbalance has been demonstrated difficulty, deploying such a learning strategy will strengthen model robustness and reliability. Our proposed approach deploys curriculum learning (*CL*) [29] that automatically adjusts the optimization process by gradually selecting training data entries from learning difficulty — learning from hard to easy samples as our optimization strategy [30]. To achieve the goal, we design two major CL modules: a difficulty measurer for assessing the difficulty of samples, and a training scheduler for determining the percentage of training data. Then we employ our CL training strategy to two tasks. Task 1 aims to predict entities from the images, and task 2 can generate a report from the images’ features and entity distribution.

*Difficulty measurer* is the core scoring function of the curriculum learning that decides which data samples should be fed to models for training. To diversify learning aspects and jointly incorporate imbalance factors, we propose a novel measurement to improve model performance over imbalance patterns. Our measurement adopts a competitive mechanism that encourages correct options with higher ranking over incorrect ones, rather than independently increasing the likelihood of correct options and decreasing the likelihood of incorrect options. This approach helps mitigate overfitting on common samples and underfitting on rare samples since it focuses on ranking of correct options rather than prediction confidence. Specifically, given a reference token *z*, vocabulary list *V*, and the prediction $\textbf{p} \in \mathcal {R}^{|V|}$, we calculate the token reference (*z*) probability ranking in the prediction $\textbf{p}$ as the following:

1 $$\begin{aligned} k = Rank(\textbf{p}, \textbf{p}[z])/ |V| \end{aligned}$$

where |*V*| is the vocabulary size. $Rank(\textbf{p}, \textbf{p}[z])$ assigns a rank to $\textbf{p}$ in descending order and identifies the position of $\textbf{p}[z]$ within this ranking. We used the *Rank*() function to inference imbalance distributions on the token level, which will give a higher preference towards tokens that are hard to predict. To avoid the biased performance evaluation in different labels and tokens, we calculate the average value in non-entity and entity tokens separately, extracted by the Radgraph [31]. *k* ranges from 0 to 1 under regularization with |*V*|. A higher value of *k* indicates that the sample is more difficult. Then, we feed the difficulty information to the next step, Training Scheduler.

*Training scheduler* aims to automatically leverage imbalance effects by selecting training samples via the difficulty measurers. Our goal is to increase the number of easier samples when the performance decreases and vice versa. According to our goal, we design our scheduler function, $c(s_{t})$ as follows:

2 $$\begin{aligned} c(s_{t}) = min(1, [1-\frac{(s_{t}-s_{t-1})}{s_{t-1}}] \times c(s_{t-1})), t \ge 1 \end{aligned}$$,

where *s* is the average performance of all training samples, measuring the model’s learning ability. *t* is the training step. Given decreasing performance as an example, $\frac{(s_{t}-s_{t-1})}{s_{t-1}}$ will be negative. During the process, the ratio $1-\frac{(s_{t}-s_{t-1})}{s_{t-1}} > 1$ will allow the model to include more easy training data than the last step $c(s_{t-1})$. When the performance increases, the scheduler feeds less easy samples to the model and reduces the over-fitting on these samples. After multiple epochs of training, harder samples receive more training iterations than easier samples. In this way, we can alleviate the challenge from imbalanced tokens and labels in the radiology report generation task. To start our curriculum learning, we record the samples’ average performance of the last two regular training epochs as $s_{0}$ and $s_{1}$, where we empirically initialize $c(s_{0})$ as 1.

### CL-Task 1

CL-Task 1 is to exploit imbalance patterns of radiology labels to generate clinically accurate reports. Entities in clinical reports play a crucial role in disease diagnosis. However, these clinical tokens often occur infrequently and are significantly underestimated during model training. Hence, we assess the accuracy of clinical entities to evaluate performance. Our intuition is that as abnormal cases contain more infrequent entities, focusing on the clinical entities may benefit the abnormal cases. If our generated reports are clinically correct, the visual extractor can accurately extract the same entities as gold entities from images.

The computing process is as follows. Given a radiology image *Img* and the corresponding report $Z =\left( z_{0}, \ldots , z_{l}\right)$ with the length *l*, we extract the features from images with a visual extractor. We use ResNet101 [32] ($f_{\mathcal {R}}$) as our visual extractor and obtain image features ($\textbf{X}$) from different convolutional channels, $\textbf{X} = f_{\mathcal {R}}({\text {Img}})$. $\textbf{X} \in \mathcal {R}^{patch\_size \times d}$, where *d* is the size of the feature vector. To predict entities distribution, we feed the feature from $\textbf{X}$ into the Entity Extractor ($f_{E}$) with parameters $W_{E} \in \mathcal {R}^{d \times |V|}$ and average the value on each patch(1st dimension),

3 $$\begin{aligned} \textbf{q} = AVG_{:1}(f_{E}(\textbf{X} | W_{E})) \end{aligned}$$

Then we obtain the entity distribution representation $\textbf{q} \in \mathcal {R}^{|V|}$. To optimize the model, we minimize Binary Cross Entropy as follows,

4 $$\begin{aligned} \mathcal {L}_{task1} = \frac{1}{|V|} \sum _{i=1}^{|V|}-\left( y_i ^* \log \left( q_i\right) +\left( 1-y_i\right) * \log \left( 1-q_i\right) \right) \end{aligned}$$

where $q_{i}$ is the prediction probability of the i-th token and $y_{i} = 1$ if i-th token is the entities. We extract the gold entities ($\textbf{e}$) by radgraph [31]. To evaluate the sample’s difficulty in this task, we input the entity distribution prediction $\textbf{q}$ into (1) and obtain $k^{task1} = \sum _{i}^{|\textbf{e}|} Rank(\textbf{q},\textbf{q}[e_{i}])/( |V| \cdot |\textbf{e}|)$.

### CL-Task 2

CL-Task 2 implements an image-to-text generation pipeline with the objective of improving the infrequent tokens prediction in reports. To generate a report containing more clinically useful information, we integrate the probability prediction of entities($\textbf{q}$) in (3) with image’s feature ($\textbf{X}$). Since $d \ne |V|$, we cannot interact $\textbf{q}$ and $\textbf{X}$ directly. To facilitate their interaction and information sharing, we employ a cross-concatenation and perform an element-wise multiplication on their cross-concatenated matrix as follows:

$$\begin{aligned} \textbf{S} = concat_{:2}(\textbf{X}, \textbf{q}) \odot concat_{:2}(\textbf{q}, \textbf{X}) \end{aligned}$$

where $\textbf{S} \in \mathcal {R}^{patch\_size \times (d + |V|)}$and $\odot$ refers to the element-wise multiplication. We empirically compare with the simple sum, dot product, and cross-modal attention network [33]; however, the element-wise multiplication achieved the best results on the validation set. We infer that the multiplication is less complex than the cross-modal attention network while keeps more vector information from the text and image modalities. Finally, we adopt a transformer structure to encode $\textbf{S}$ and generate i-th token probability distribution $\mathbf {P_{i}}$ from encoding feature $\textbf{S}$ and i-th token, $\mathbf {{P}_{i}} = f_{{\mathcal {T}}}(\textbf{S}, z_{i-1})$. To optimize the model, we minimize negative log-likelihood loss (NLL) as follows,

5 $$\begin{aligned} \mathcal {L}_{task2} = -\sum _{i}^{l} \log \left( \mathbf {P_{i}}\right) \end{aligned}$$

We can access the sample’s difficulty with $\mathbf {P_{i}}$ by (1), $k^{task2} \!=\! \sum _{i}^{l} Rank(\textbf{P}_{i},\textbf{P}_{i}[z_{i}])/( |V| \cdot l)$.

**Algorithm 1:**
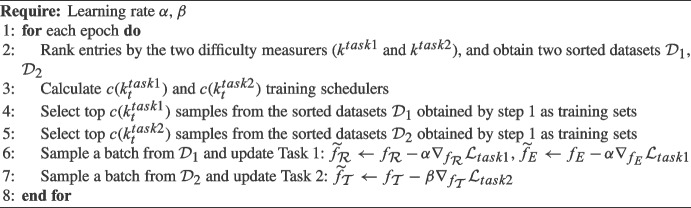
Optimization Process of JIMA.

### CL-Joint Optimization

We propose a joint optimization approach to integrate two tasks. Algorithm 1 summarizes the overall optimization process of our approach. We set the learning rate of task 1 as $\alpha$, and $\beta$ refers to the learning rate of task 2. In each training step, we sample different data for different tasks, and each task focuses on optimizing its own module of the models. For example, we update the visual extractor ($f_{\mathcal {R}}$) and the entity extractor ($f_{E}$) in task 1. Next, we freeze the parameters of the visual extractor and the entity extractor and update the parameters of the transformer ($f_{\mathcal {T}}$) specifically for task 2. Our optimization approach integrates with curriculum learning to tailor joint imbalance learning for each module ($f_\mathcal {R}$, $f_{E}$, $f_\mathcal {T}$). Curriculum learning empowers the model to concentrate on optimizing hard samples while mitigating the risk of overfitting to easier samples. The joint optimization scheme facilitates each task to manage different module parameters optimization and learn transferable knowledge from the simpler to more complex tasks. As a result, all modules collaborate to enhance error reduction from previous tasks.

## Experiments

We design our experiments to evaluate performance on both regular and imbalanced settings via automatic and human evaluations. The automatic evaluation includes NLG-oriented and clinical-correctness metrics. NLG-oriented metrics measure the similarity between generated and reference reports. Clinical correctness and human evaluation belong to factually oriented metrics and domain-specific evaluation methods. To be consistent with our baselines [10, 13, 19], we utilize the F1 CheXbert [34] for the clinical-correctness metrics. The experiments compare our proposed approach (JIMA) and the state-of-the-art baselines. Two of our five baselines (CMM + RL & RRG) are designed to solve label imbalance by improving the abnormal findings generation. We conduct ablation and case analyses to fully understand the capabilities of our proposed approach. We include more implementation details and hyperparameter settings in Appendix 2.1.

### Baselines

To examine the validity of our method, we include five state-of-the-art baselines under the same experimental settings: R2Gen [19], CMN [26], WCL [25], CMN + RL [20], RRG [23], TIMER [10], and RGRG [35] — and obtain from their open-sourced code repositories.

**R2Gen** [19] is a transformer-based model with ResNet101 [32] as the visual extractor. To capture some patterns in medical reports, R2Gen proposes a relational memory to enhance the transformer so that the model can learn from the patterns’ characteristics. Furthermore, R2Gen deploys a memory-driven conditional layer normalization to the transformer decoder, facilitating the incorporation of the previous step generation into the current step.

**CMN** [26] is a novel extension to the transformer architecture that facilitates the alignment of textual and visual modalities. The cross-modal memory network records the shared information of visual and textual features. The alignment process is carried out via memory querying and responding. The model maps the visual and textual features into the same representation space in memory querying and learns a weighted representation of these features in memory responding.

**WCL** [25] utilizes the R2Gen framework and incorporates a weakly supervised contrastive loss. Specifically, WCL leverages the contrastive loss to enhance the similarity between a given source image and its corresponding target sequence. Furthermore, the model enhances its ability to learn from difficult samples by assigning more weights to instances sharing common labels.

**CMM + RL** [20] is a cross-modal memory-based model with reinforcement learning for optimization. CMM + RL designs a cross-modal memory model to align the visual and textual features and deploy reinforcement learning to capture the label imbalance between abnormality and normality. The author uses BLEU-4 as a reward to guide the model to generate the next word from the image and previous words.

**RRG** [13, 23] aims to generate clinically correct reports by weakly supervised learning of the entities and relations from reports. RRG is a BERT-based model with Densenet-121 [36] as a visual extractor. RRG leverages RadGraph [31] to extract the entities and relation labels in a report. RRG utilizes reinforcement learning to optimize the model. The reward assesses the consistency and completeness of entities and the relation set between generated reports and reference radiology reports. RRG addresses label imbalance issues by maximizing the reward of predicting more complicated entities and relations in abnormal samples.

**TIMER** [10] aims to decrease the over-fitting of frequent tokens by introducing an unlikelihood loss to punish the error on these tokens. The tokens set of unlikelihood loss is automatically adjusted by maximizing the average F1 score on different frequency tokens.

**RGRG** [35] adopts GPT2 as the language generation model and generates a report based on the localized visual features of anatomical regions, which are extracted by an object detection. This baseline experiment was specifically carried out on the MIMIC-CXR dataset, as the IU X-ray dataset lacks anatomical region information, resulting in the inability to train an object detection module effectively.

### Imbalance Setting

We evaluate model robustness under token and label imbalance settings and present results in Sects. 6.2 and 6.3. For token imbalance, we compare F1 scores of frequent and infrequent tokens separately. We introduce three different scales to define frequency token sets, 1/4, 1/6, and 1/8 respectively. The splits define the top 1/4, 1/6, and 1/8 vocabulary as frequent tokens and the rest vocabulary as infrequent tokens. The setting is to demonstrate the effectiveness of our approach in adapting token imbalance. For label imbalance, we divide our samples into a binary category, normal and abnormal.

**Table 2:** Overall performance

Dataset	Model	NLG metrics						CE metrics
		BLEU-1	BLEU-2	BLEU-3	BLEU-4	METEOR	ROUGE-L	F1
IU X-ray	R2Gen	48.80	31.93	23.24	17.72	20.21	37.10	63.62
	CMN	45.53	29.50	21.47	16.53	18.99	36.78	64.83
	WCL	44.74	29.30	21.49	16.79	20.45	37.11	49.24
	CMM + RL	49.40	30.08	21.45	16.10	20.10	38.40	40.79
	RRG	49.96	31.44	22.11	17.05	18.81	33.46	49.10
	TIMER	49.34	32.49	23.84	18.61	20.38	38.25	94.52
	JIMA (Ours)	**50**.**50**	**33**.**12**	**24**.**15**	**18**.**88**	**21**.**16**	**38**.**56**	**96**.**58**
	$\overline{\Delta }$ (%)$^\textrm{B}$	5.49	7.74	8.65	10.44	6.86	4.86	72.10
	$\hat{\Delta }$ (%)$^\textrm{B}$	2.35	1.93	1.30	1.45	3.82	0.81	2.18
MIMIC-CXR	R2Gen	35.42	21.99	14.50	10.30	13.75	27.24	54.60
	CMN	35.60	21.41	14.07	9.91	14.18	27.14	50.50
	WCL	37.30	23.13	15.49	10.70	14.40	27.39	55.58
	CMM+RL	35.35	21.80	14.82	10.58	14.20	27.37	65.43
	RRG	37.57	19.78	15.87	9.56	14.77	26.81	62.20
	TIMER	38.30	22.49	14.60	10.40	14.70	28.00	75.86
	RGRG	30.7	20.59	14.10	10.18	15.43	24.03	80.28
	JIMA (Ours)	**41**.**37**	**24**.**83**	**16**.**72**	**11**.**20**	**16**.**75**	**30**.**15**	**81**.**25**
	$\overline{\Delta }$(%)$^\textrm{B}$	16.26	15.24	13.34	9.59	15.73	12.52	31.29
	$\hat{\Delta }$(%)$^\textrm{B}$	8.02	10.40	14.52	7.69	13.94	7.68	1.21

$\overline{\Delta }$ is the averaged percentage improvements over baselines, and the $\hat{\Delta }$ refers to the percentage improvements over the state-of-the-art model. The evaluation includes both generation (NLG) and clinical-correctness (CE) metrics, where bold numbers indicate the best performance

## Results and Analysis

In this section, we present overall performance and report results of imbalance evaluations and include an ablation analysis and a case study. Generally, JIMA outperforms the state-of-the-art baselines by a large margin, especially under imbalance settings. Our qualitative studies show our method can achieve more clinically accuracy and generate more precisely clinical terms.

### Overall Performance

Table 2 presents the performance of JIMA by NLG and clinical-correctness metrics. JIMA outperforms baseline models (both imbalance and regular methods) on BLEU scores by a large margin, confirming the validity of selecting training samples by our curriculum learning method. The approach enables the model to learn multiple times from the samples with lower BLEU-4, resulting in a better performance compared to the baseline models. For example, JIMA shows an improvement of 16.59% on average for IU X-ray and 16.28% for MIMIC-CXR. We infer this is as our tasks 1 and 2 jointly work to improve the token and label imbalanced problem.

Second, our model achieves the best performance in F1 of the clinical metric, which indicates that task 1 (Sect. 4.1) can enable the model to put more attention on difficult samples with lower F1 scores. Additionally, our method promotes clinical token prediction as performance on infrequent tokens and medical terms has been improved. For example, our generation significantly outperforms the baselines on F1 score by 72.10% on IU X-ray and 31.29% on the MIMIC-CXR average. CMN $+$ RL performs better than other baselines on IU X-ray but not on MIMIC-CXR. JIMA maintains a stable performance on both IU X-ray and MIMIC-CXR. We infer this as our joint imbalance adaptation can yield more improvements.

### Token Imbalance

Table 3 compares high- and low-frequent tokens F1 in different ratio splits. Our method consistently outperforms baselines in the low-frequent tokens across frequency splits ($\frac{1}{4}, \frac{1}{6}$, and $\frac{1}{8}$) on IU X-ray and MIMIC-CXR. While RRG and CMN + RL approaches have adapted label imbalance, the approaches may not be able to adapt the token imbalance. Our approach achieves better performance on the token imbalance. Generating rare tokens with accuracy remains a difficult task despite the high performance achieved on frequent tokens. Common tokens are prone to overfitting while rare tokens are predicted with less precision. For example, the 0.00 score by R2GEN on 3/4 split of the MIMIC-CXR vocabulary. Performance imbalance can deteriorate the clinical correctness of generated reports as medical terminologies are usually infrequent. Nonetheless, our joint imbalance adaptation approach has shown considerable improvements in this area, indicating a promising direction to enhance the robustness of radiology report generation, a critical clinical task.

**Table 3:** Results on high- and low-frequent tokens with three ratio splits

		IU X-ray		MIMIC-CXR	
Ratio	Method	infreq	freq	infreq	freq
1/8	R2GEN	4.46	**62**.**73**	2.52	52.01
	CMN	5.88	55.86	2.23	45.60
	WCL	5.29	60.23	2.91	48.60
	CMN + RL	5.19	49.36	0.21	23.64
	RRG	7.28	41.94	2.50	43.57
	TIMER	13.23	61.89	3.15	52.66
	RGRG	-	-	0.22	31.33
	JIMA (ours)	**14**.**87**	62.55	**3**.**58**	**53**.**06**
1/6	R2GEN	2.80	61.62	2.02	49.86
	CMN	5.75	65.12	0.85	52.02
	WCL	3.72	59.26	2.13	47.88
	CMN + RL	5.19	49.36	0.14	23.36
	RRG	4.55	40.46	2.09	43.56
	TIMER	5.93	67.79	2.02	51.72
	RGRG	-	-	0.26	30.66
	JIMA (ours)	**10**.**52**	**68**.**82**	**2**.**83**	**52**.**32**
1/4	R2GEN	1.16	59.98	0.00	48.77
	CMN	2.60	63.92	0.33	51.09
	WCL	1.50	56.83	0.30	46.95
	CMN + RL	5.19	49.36	0.07	23.05
	RRG	2.04	38.84	0.39	41.45
	TIMER	8.66	64.00	0.58	51.39
	RGRG	-	-	0.20	29.56
	JIMA (ours)	**9**.**77**	**66**.**23**	**0**.**94**	**51**.**92**

We measured the model performance on frequent and infrequent tokens by F1 score

### Label Imbalance

We report NLG evaluations on label imbalance (normal vs. abnormal) in Table 4. JIMA significantly outperforms baseline models both on normal and abnormal splits, which demonstrates its effectiveness under label imbalance. JIMA also performs better than the label imbalance methods, RRG and CMM+RL, indicating that the joint imbalance adaptation is a promising direction to improve model robustness. It is worth noting that models generally perform better on normal samples than on abnormal ones. We infer this for two reasons: (1) abnormal reports contain more infrequent medical tokens and (2) abnormal reports are longer, as discussed in Sect. 3. JIMA shows more improvements on abnormal samples over baselines while maintains a similar performance on samples with normal labels. The observations suggest that our approach can successfully learn from lengthier documents with more medical tokens.

**Table 4:** Label imbalance evaluation with binary label types, normal and abnormal

Dataset	Label	Model	BLEU-1	BLEU-2	BLEU-3	BLEU-4	METEOR	ROUGE-L
IU X-ray	Normal	R2Gen	50.50	34.91	25.86	20.93	23.66	40.56
		CMN	47.42	32.80	25.25	18.72	20.51	38.69
		WCL	49.74	35.44	28.02	18.71	26.88	42.09
		CMM+RL	51.68	36.65	21.99	19.47	24.53	40.05
		RRG	50.03	33.76	24.81	19.89	20.43	34.39
		TIMER	51.83	32.43	33.71	20.19	24.43	39.39
		JIMA (ours)	**52**.**65**	** 37.06**	**28**.**39**	**21**.**56**	**27**.**20**	**42**.**33**
	Abnormal	R2Gen	42.67	27.86	18.47	12.35	15.04	30.10
		CMN	35.09	21.42	14.97	11.32	14.36	29.85
		WCL	32.31	19.93	13.87	10.50	13.81	30.37
		CMM+RL	38.09	25.42	11.17	15.09	13.13	27.64
		RRG	43.38	23.44	10.02	15.58	12.43	31.52
		TIMER	44.25	26.73	15.28	10.76	15.43	33.26
		JIMA (ours)	**45**.**41**	**27.95**	**19**.**15**	**15**.**68**	** 16.36**	**34**.**59**
MIMIC-CXR	Normal	R2Gen	40.42	26.76	19.75	15.60	17.58	32.02
		CMN	41.42	27.80	20.25	15.72	17.51	33.69
		WCL	39.74	25.44	18.02	13.71	16.88	32.09
		CMM+RL	17.50	10.11	6.83	14.99	8.05	19.10
		RRG	38.78	21.63	18.04	12.09	18.27	27.56
		TIMER	40.33	27.53	19.88	14.87	17.47	33.08
		RGRG	32.09	22.67	16.40	12.30	18.26	27.28
		JIMA (ours)	** 41.79**	**27.87**	**20**.**49**	**16**.**00**	**17**.**93**	**33**.**87**
	Abnormal	R2Gen	33.97	19.31	12.07	10.17	10.98	26.82
		CMN	33.00	19.44	10.02	8.73	10.21	25.16
		WCL	34.56	22.45	14.63	10.26	12.43	26.87
		CMM+RL	27.74	10.87	5.18	3.43	6.11	16.08
		RRG	17.47	9.71	5.78	3.74	8.37	17.59
		TIMER	35.66	21.83	14.25	14.87	9.84	26.77
		RGRG	30.54	20.34	13.82	9.92	15.13	23.66
		JIMA (ours)	**37**.**81**	**22**.**46**	**15**.**26**	**10**.**28**	**14**.**56**	**27**.**38**

### Ablation Analysis

**Fig. 4:**
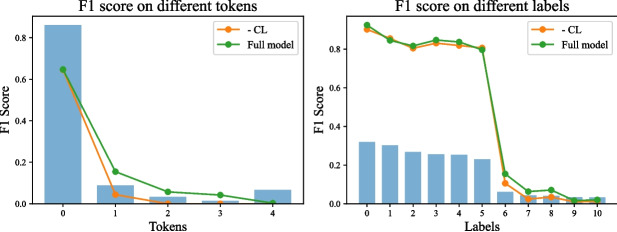
Ablation analysis for JIMA performance comparison with and without curriculum learning across various labels and tokens frequencies

In this section, we carry out ablation experiments to analyze the impact of our curriculum learning approach on tokens and labels with different frequencies. To investigate the performance across different tokens, we categorize tokens into five groups based on their frequency, with “0” representing the most frequent tokens and “4” representing the least frequent tokens. Each group contains an equal number of tokens. In order to compare the performance across different labels, we present their performance individually. We conduct our ablation experiments on the MIMIC-CXR dataset, and the results are depicted in Fig. 4.

**Table 5:** Human evaluation on the state-of-the-art baseline and our approach

Dataset	Label	CMM+RL	Same	JIMA (Ours)
IU X-ray	Normal	6 — 7	12 — 7	**6 — 10**
	Abnormal	4 — 4	10 — 5	**12 — 13**
MIMIC-CXR	Normal	6 — 7	15 — 7	**7 — 11**
	Abnormal	5 — 6	10 — 7	**7 — 16**
Overall	Normal	12 — 14	27 — 14	**13 — 21**
	Abnormal	9 — 10	20 — 12	**19 — 29**
	All	21 — 24	47 — 26	**32 — 50**

The baseline does not consider the imbalance effects. To better illustrate the varying performance on the labels, we report performance conditioning on the normal and abnormal reports. “Same” means the experts vote the same for the generated reports

First, we notice that removing curriculum learning does not result in a significant detrimental impact on highly frequent tokens and labels. For instance, the performance is comparable in the “0” token group and the “0–5” label groups. Curriculum learning empowers the model to allocate increased attention to challenging samples, thereby reducing the likelihood of predictions on highly frequent samples. However, our curriculum learning strategy selects training samples based on the ranking of the correct answers. Therefore, despite the reduced probability of the correct answer, the ranking remains unchanged. For example, the correct option still holds the highest estimation). As a result, our curriculum learning approach does not diminish the performance on highly frequent samples. Next, our curriculum learning approach significantly enhances performance primarily on moderately frequent samples. The average improvement amounts to 6.49% in the “1–3” token group and 2.58% in the “6–10” label group. However, our method exhibits limitations in enhancing the performance of exceedingly rare tokens. Notably, the model struggles to predict tokens in the “4” group.

### Human Evaluation

To verify the factual correctness, we invite two health professionals to perform the evaluation. First, we randomly select 50 test instances per data from IU X-ray and MIMIC-CXR, respectively. We choose CMM+RL as our targeting comparison, as the model is the best-performing baseline by automatic metrics. In evaluation, we show the X-ray images, corresponding ground truth reports, and two generated reports (one from our model and the other from CMM+RL) to the expert without disclosing their sources. The experts selected a better description from two candidate reports or chose the “Same” option if both reports are of similar quality.

We present our human evaluation results in Table 5, which shows a consistent result with automatic evaluation results. Generally, JIMA outperforms the baseline with 11 reports in total. Notably, our approach exhibits significant improvements in abnormal samples. Even though JIMA has only one more vote than the baseline in normal samples, our model secures ten more votes in abnormal samples. This is because abnormal samples have lengthier reports on average and encompass more medical entities, indicating that our approach generates more clinically precise reports. Furthermore, our human evaluation is consistent with the automated evaluation results shown in Table 2.

### Case Study

**Fig. 5:**
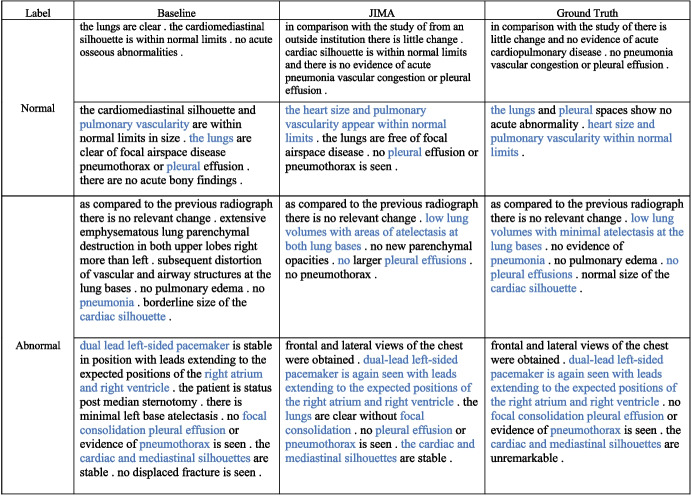
Qualitative comparison between JIMA and CMM+RL. We highlight correct predictions of pathological and anatomical entities in blue color

To verify our model’s effectiveness in generating clinically correct descriptions, we perform a case study in this section and present the result in Fig. 5. We select four samples from IU X-ray and MIMIC-CXR datasets and compare the normal and abnormal samples’ performance separately. The correct pathological and anatomical entity predictions are remarked in blue color. Generally, our predictions cover more than 90% entities in reference reports. Compared to normal samples, abnormal samples have longer descriptions and contain more complex entities. These entities usually are rare in corpus and suffer under-fitting from models. Therefore, models underperform in abnormal samples. However, JIMA can capture most of the entities in all kinds of samples and achieve similar performance in both normal and abnormal samples, which proves our model’s effectiveness in improving the factual completeness and correctness of generated radiology reports.

## Conclusion

In this study, we have examined the imbalance effects on the radiology report generation models from two imbalance factors, label and token. We developed the Joint Imbalance Adaptation (JIMA) model to encounter the multifaceted imbalance challenge. JIMA takes a novel curriculum learning approach to jointly learn imbalance patterns of the radiology token and image label by two modules, difficulty measurer and training scheduler. Extensive experiments, ablation analysis, and human evaluations show that JIMA leads to improvements over the existing state-of-the-art baselines between [0.81%, 3.82%] on IU X-ray and [1.21%, 14.52%] on MIMIC-CXR. Our approach also promotes model robustness in handling token and label imbalance, as shown in Tables 3 and 4. Particularly, our ablation analysis shows that JIMA does not significantly reduce performance on highly frequent tokens and labels, yet significantly improves performance for moderately frequent samples, and still exhibits some limitations in enhancing the performance of the rarest tokens. This study makes *a unique contribution* to the radiology report generation that jointly considers multiple imbalance factors via curriculum learning. Our future work will focus on refining the JIMA approach to address the limitations highlighted in our ablation study, including low model performance on the exceedingly rare tokens. Further exploration will also include a deeper analysis of other imbalance factors, such as different demographic groups.

## Limitations

Limitations should be fully acknowledged before fully interpreting this study, as no research can be fully perfect. **Rare tokens.** Our approach has improved the model performance on the rare tokens but still keeps relatively lower F1 scores than the frequent tokens. For example, the JIMA model achieves F1 scores of 14.87 on the infrequent tokens versus 62.55 on the frequent tokens on the 1/8 ratio split of the IU-Xray. Our ongoing work will explore if other imbalance learning approaches (e.g., data augmentation [24]) or combining other approaches with our JIMA can achieve better performance. **Evaluation.** We are aware of *other evaluation metrics*, such as RadGraph [31] and CheXpert [37]. However, additional metrics may only be applicable to the MIMIC-CXR or have overlapped with our existing method, such as CheXpert and CheXbert [34]. We have included diverse metrics, including NLG, clinical correctness, and human evaluations. To keep consistency with our state-of-the-art baselines, we utilize a similar evaluation schema. Having consistent observations between our human and automatic evaluations may also prove our evaluation’s validity.

## Data Availability

The data that support the findings of this study are derived from the publicly available MIMIC-CXR and IU-Xray datasets. MIMIC-CXR: The MIMIC-CXR (Medical Information Mart for Intensive Care) dataset is available through the PhysioNet repository. More information on accessing MIMIC-CXR can be found at https://physionet.org/content/mimic-cxr/2.0.0/. IU-Xray: The IUXray dataset, which consists of chest X-ray images and associated radiology reports, is available from the Open Access Biomedical Image Search Engine (OpenI) provided by the U.S. National Library of Medicine. The dataset can be accessed at https://openi.nlm.nih.gov/faq#collection. These datasets are publicly available to researchers subject to the respective data use agreements and ethical guidelines. Any additional data generated and analyzed during the current study are available from the corresponding author on reasonable request.

## Appendix 1. Data

We extract labels of each data entry and follow baseline studies [19, 20, 26] to preprocess the report documents to ensure comparisons under the same settings. In order to ensure data format consistency, we include and infer two primary labels of radiology reports, normality and abnormality. To obtain labels for IU X-ray, we build a supervised classifier using BioBert-PubMed200kRCT [38] to extract the binary labels on the Medical Subject Heading (MESH)^2^ and RadLex^3^ labels (normal and abnormal). To obtain labels for MIMI-CXR, we utilize CheXbert [34] to extract the binary categories, disease types, and “no finding.” We define “no finding” as normality and disease types as abnormality. In this study, we conducted text preprocessing by utilizing the Natural Language Toolkit (NLTK) [39] to lowercase and tokenize documents. Furthermore, we removed redundant spaces, empty lines, serial numbers, and punctuation marks from the documents.

### 1.1. Ethic, Privacy, and IRB

We follow data agreement and training to access the two radiology report datasets. To protect user privacy, we ensure proper data usage and experiment with de-identified data. Our experiments do not store any data and only use available multimodal entries for research demonstrations. Due to privacy and ethical considerations, we will not release any clinical data associated with patient identities. Instead, we will release our code and provide detailed instructions to replicate our study. This study only uses publicly available and de-identified data. Our study focuses on computational approaches and does not collect data from human subjects. Our institutional IRB determines that IRB approval is not required for this study.

## Appendix 2. Experiment

### 2.1 Implementation Details

In our model architecture, we set the transformer structure with 3 layers and 8 attention heads, 512 dimensions for hidden states. The memory-driven model is a single-layer GRU network with a hidden size equal to vocabulary size. We set the $\alpha$ learning rate as $4e-4$ and $\beta$ learning rate as $1e-5$ and decay them by a 0.8 rate per epoch for all datasets. The pre-training epoch is 30 in IU X-ray and 10 in MIMIC-CXR. Then we adopt curriculum learning to optimize our pre-trained model. The maximum training epoch is 70 for the IU X-ray and 50 for the MIMIC-CXR datasets. We keep the learning rate the same as in the pre-trained stage.

For all baselines, we set the maximum training epoch as 100 and 60 for IU X-ray and the MIMIC-CXR datasets, respectively. Also, we use the same preprocessing, optimizer, batch size, maximum length of training data, sampling method, and machine learning framework in all experiments. Specifically, we optimize models by ADAM [40] with 16 batch sizes. The maximum length of training data is 60. In the test stage, we generate tokens by beam search [41] with 3 beam sizes for all experiments. All implementations are on PyTorch [42]. In implementing baselines, we keep all the model architecture and optimization parameters the same as in their papers. In R2Gen, CMN, and RRG, we generate reports by using the code and the pre-trained models published by the authors. For the other baselines (WCL & CMM+RL & TIMER), we use the released code to train and generate reports.

We personalize the following setting in baselines. In WCL, we use the basic contrastive learning loss without assigning a hardness weight to different samples in IU X-ray dataset. Because the file measuring the similarity among different samples is inaccessible. We set the contrastive embedding size as 256 and the weight of contrastive loss is 0.2. In CMM + RL, the reinforcement learning reward is based on evaluation metrics and we select BLEU-4 in this case.

### 2.2. Evaluation Metrics

**Automatic Evaluation** includes seven evaluation methods from two major categories, *NLG* and *Clinical metrics*. We first evaluate our model and the baseline models on *natural language generation (NLG) metrics*, including BLEU (−1, −2, −3, and −4) [27], METEOR [43] and ROUGE-L [44]. BLEU score measures the precision of prediction with a penalty for the reference-to-prediction length ratio. METEOR computes the harmonic mean of unigram precision and recall. Unlike BLEU, which considers only single words, METEOR incorporates a penalty to account for the importance of word order. ROUGE-L takes into account sentence-level structure similarity naturally and identifies the longest co-occurring in sequence n-grams automatically. *Clinical metrics* is a domain-specific evaluation method to measure the factual completeness and consistency of generated reports. We use CheXbert [34] to extract the labels of ground truth and prediction and evaluate clinical efficacy (CE) metrics by F1. We do not present clinical F1 score in the label imbalance experiment since we can not access recall in separate normal and abnormal sample sets.

## Footnotes

1 Clinical reports are also much longer than general-domain image captions, such as MS-COCO [6].

## Code Availability

The code that implements the method described in this study is publicly available at https://github.com/trust-nlp/JIMA/. This repository includes all necessary scripts, tools, and documentation to replicate the results and analyses presented in this paper.
